# Syntaxin 4 regulates the surface localization of a promyogenic receptor Cdo thereby promoting myogenic differentiation

**DOI:** 10.1186/s13395-015-0052-8

**Published:** 2015-09-07

**Authors:** Miran Yoo, Bok-Geon Kim, Sang-Jin Lee, Hyeon-Ju Jeong, Jong Woo Park, Dong-Wan Seo, Yong Kee Kim, Hoi Young Lee, Jeung-Whan Han, Jong-Sun Kang, Gyu-Un Bae

**Affiliations:** Research Center for Cell Fate Control, College of Pharmacy, Sookmyung Women’s University, Seoul, 140-742 Republic of Korea; Department of Molecular Cell Biology, Sungkyunkwan University School of Medicine, Samsung Biomedical Research Institute, Suwon, 440-746 Republic of Korea; Research Center for Epigenome Regulation, School of Pharmacy, Sungkyunkwan University, Suwon, Republic of Korea; College of Pharmacy, Dankook University, Cheonan, 330-714 Republic of Korea; Department of Pharmacology, Myunggok Medical Research Institute, College of Medicine, Konyang University, Daejeon, Republic of Korea

**Keywords:** Syntaxin 4, Cdo, Myogenic differentiation, p38MAPK, Cell surface localization

## Abstract

**Background:**

Syntaxins are a family of membrane proteins involved in vesicle trafficking, such as synaptic vesicle exocytosis. Syntaxin 4 (Stx4) is expressed highly in skeletal muscle and plays a critical role in insulin-stimulated glucose uptake by promoting translocation of glucose transporter 4 (GLUT4) to the cell surface. A cell surface receptor cell adhesion molecule-related, down-regulated by oncogenes (Cdo) is a component of cell adhesion complexes and promotes myoblast differentiation via activation of key signalings, including p38MAPK and AKT. In this study, we investigate the function of Stx4 in myoblast differentiation and the crosstalk between Stx4 and Cdo in myoblast differentiation.

**Methods:**

The effects of overexpression or shRNA-based depletion of Stx4 and Cdo genes on C2C12 myoblast differentiation are assessed by Western blotting and immunofluorescence approaches. The interaction between Cdo and Stx4 and the responsible domain mapping are assessed by coimmunoprecipitation or pulldown assays. The effect of Stx4 depletion on cell surface localization of Cdo and GLUT4 in C2C12 myoblasts is assessed by surface biotinylation and Western blotting.

**Results:**

Overexpression or knockdown of Stx4 enhances or inhibits myogenic differentiation, respectively. Stx4 binds to the cytoplasmic tail of Cdo, and this interaction seems to be critical for induction of p38MAPK activation and myotube formation. Stx4 depletion decreases specifically the cell surface localization of Cdo without changes in surface N-Cadherin levels. Interestingly, Cdo depletion reduces the level of GLUT4 and Stx4 at cell surface. Consistently, overexpression of Cdo in C2C12 myoblasts generally increases glucose uptake, while Cdo depletion reduces it.

**Conclusions:**

Stx4 promotes myoblast differentiation through interaction with Cdo and stimulation of its surface translocation. Both Cdo and Stx4 are required for GLUT4 translocation to cell surface and glucose uptake in myoblast differentiation.

**Electronic supplementary material:**

The online version of this article (doi:10.1186/s13395-015-0052-8) contains supplementary material, which is available to authorized users.

## Background

Skeletal myoblast differentiation is a well-coordinated multistep process that involves cell cycle withdrawal, expression of muscle-specific genes, and formation of multinucleated myofibers by cell fusion [1]. Two groups of transcription factors, the myogenic determination factors (Myf5, MyoD, Myogenin, and MRF4) and the myocyte enhancer factor 2 (MEF2), are central for the coordination of myogenesis [2–4]. The expression and activities of these transcription factors are tightly regulated to ensure efficient myogenic differentiation and to maintain the differentiated state of cells. Once activated, these transcription factors regulate numerous downstream target genes to initiate myogenic differentiation and reinforce each other’s expression, resulting in a positive feedback network that amplifies and maintains the myogenic phenotype [5, 6].

Cell-cell adhesion between muscle precursors plays a crucial role in myoblast differentiation. A cell surface receptor cell adhesion molecule-related, down-regulated by oncogenes (Cdo) appears to be a critical component that integrates cell-contact-mediated signals from the cell surface into the myogenic regulatory network [7, 8]. Cdo forms a multiprotein complex with other cell adhesion molecules including N-Cadherin, Gas1, Boc, and neogenin/netrin-3, resulting in the promotion of myogenesis [9–12]. The depletion of Cdo in myoblasts shows impaired myogenic differentiation, and Cdo-deficient mice display delayed skeletal muscle development [7, 13]. In contrast, overexpression of Cdo in C2C12 cells enhances myoblast differentiation. The promyogenic function of Cdo involves a coordinated activation of p38 mitogen-activated protein kinase (p38MAPK) and AKT via association with scaffold proteins, JLP and Bnip2 for p38MAPK [13, 14] and APPL1 for AKT [15]. The level of Cdo protein, presumably at the cell membrane, appears to be critical for initiation of promyogenic signaling pathways; however, it is still unclear how the activity of the Cdo protein at the cell membrane is regulated.

Membrane fusion is an obligatory event in intracellular membrane trafficking and physically merges two lipid bilayers of separate compartments allowing content mixing [16]. Soluble *N*-ethylmaleimide-sensitive factor (NSF) attachment protein receptor proteins (SNAREs) play a key role in intracellular membrane fusion events and have been divided into vesicle-membrane SNAREs (v-SNAREs) and target-membrane SNAREs (t-SNAREs) based on their subcellular localization [17]. Syntaxin 4 (Stx4) is a member of t-SNAREs and expressed highly in various tissues, including the skeletal muscle, and plays a critical role in glucose uptake in response to insulin by delivery of glucose transporter 4 (GLUT4) to the cell membrane in skeletal muscle and adipose tissue [18, 19]. In addition, Stx4 has been shown to regulate glucose-stimulated insulin secretion in beta cells [20, 21]. The physiological importance of Stx4 in the glucose uptake of skeletal muscle and whole body metabolism has been shown by studies with knockout and transgenic mice [19, 22]. Stx4 heterozygous mice display an insulin resistance with reduction of glucose uptake specifically in the skeletal muscle, without alterations in adipose tissue and liver [22]. Conversely, Stx4 transgenic mice exhibit enhanced glucose uptake and insulin-induced GLUT4 translocation to the cell membrane of the skeletal muscle [19]. The complete ablation of the Stx4 gene in mice causes early embryonic lethality before embryonic day 7.5 [23]. Thus, whether Stx4 plays any role in myogenesis is still unclear. The facts that GLUT4 expression and activity increase during myoblast differentiation [24] and the fusion of myoblasts into multinucleated myotubes is a critical step for efficient differentiation prompt us to examine the role of Stx4 in myoblast differentiation, especially in regulation of a promyogenic surface receptor Cdo.

We report here that Stx4 expression is upregulated upon induction of myoblast differentiation. Overexpression of Stx4 in C2C12 myoblasts increases myogenic differentiation via regulation of p38MAPK activity, whereas Stx4 depletion in C2C12 cells by small hairpin RNA (shRNA) decreases myogenesis. Stx4 and Cdo interact physically in differentiating myoblasts, and this interaction is mediated by the t-SNARE domain of Stx4, which is critical for the promyogenic function of Stx4. Stx4 depletion leads to declined levels of the cell surface resident Cdo without changes in the level of N-Cadherin, another Cdo-interacting protein. Interestingly, Cdo depletion affected the membrane translocation of GLUT4 and interaction of Stx4 with GLUT4, without altered total protein levels. Taken together, Stx4 promotes myogenic differentiation by binding to a promyogenic receptor Cdo and regulating its cell surface translocation thereby activating downstream p38MAPK pathway.

## Methods

### Cell culture and expression vectors

Myoblast C2C12 cells, primary myoblasts, embryonic fibroblast 10T1/2 cells, and embryonic kidney 293T cells were cultured as described previously [1]. To induce differentiation of C2C12 myoblasts, cells at near confluence were changed from Dulbecco modified Eagle’s medium (DMEM) containing 15 % fetal bovine serum (FBS; growth medium, GM) to DMEM containing 2 % horse serum (HS; differentiation medium, DM), and myotube formation was observed at 2 or 3 days of differentiation. The efficiency of myotube formation was quantified by a transient differentiation assay as previously described [1]. To generate C2C12 cells that stably overexpress Cdo, Stx4, mutant forms of Stx4, or shRNAs against Stx4 or Cdo, cells were transfected with the indicated expression vectors and Lipofectamine 2000 (Invitrogen, Carlsbad, CA), and cultures were selected in puromycin-containing medium. Five different Stx4 shRNAs obtained from Sigma-Aldrich (St. Louis, MO) were screened for their effectiveness by transfection into 293T cells. From among them, the following sequences were chosen based on the strongest knockdown effect and reproducibility: shStx4#1, 5′-CCGGGAGAGACAGAGACCCAGCTTTCTCGAGAAAGCTGGGTCTCTGTCTCTCTTTTTG-3′; shStx4#2, 5′-CCGGGAGTCCTGTCCCAGCAATTTGCTCGAGCAAATTGCTGGGACAGGACTCTTTTTG-3′. For the Stx4 deletion mutation study, the mouse Stx4 gene was amplified by Reverse Transcription Polymerase Chain Reaction (RT-PCR) of mRNAs purified from human embryonic kidney fibroblast cells. Full-length Stx4 (aa 1-299) and deletion forms of Stx4 (Stx4Δ1-153, Stx4Δ154-194, and Stx4Δ195-262) were inserted into mammalian expression vector pcDNA-myc and puroBABE-GFP-S, respectively. Hindlimb and satellite cells isolated from *Cdo*^+/+^ and *Cdo*^−/−^ mice were cultured as described previously [25]. Cells were grown in F10 medium containing 20 % FBS and basic fibroblast growth factor (bFGF; 100 ng/ml).

### Western blot analysis and immunoprecipitation

Western blot analysis was performed as previously described [26]. Briefly, cells were lysed in cell extraction buffer (10 mM Tris-HCl, pH 8.0, 150 mM NaCl, 1 mM EDTA, 1 % Triton X-100) containing complete protease inhibitor cocktail (Roche Diagnostics, Indianapolis, IN), and Sodium Dodecyl Sulfate - Polyacrylamide Gel Electrophoresis (SDS-PAGE) was performed. The primary antibodies used were anti-Stx4 (sc-101301), anti-MyoD (sc-32758), anti-Myogenin (sc-12732), anti-myc (sc-40), anti-GLUT4 (sc-53566, Santa Cruz Biotechnology, Santa Cruz, CA), anti-troponin T (SAB2102501), anti-pan-Cadherin (c3678, Sigma-Aldrich, St Louis, MO), anti-p-p38 (9211), anti-p38 (9212), anti-phospho-AKT (p-AKT; 9271), anti-AKT (9272, Cell Signaling Technology, Beverly, MA), anti-GFP (A11120, Invitrogen), anti-Cdo (AF2429, R&D Systems, Minneapolis, MN), and anti-myosin heavy chain (MHC) (MF20: Developmental Studies Hybridoma Bank, Iowa, IA). For immunoprecipitation assay, 293T cells were transfected with a combination of Cdo and either myc-tagged Stx4 or S-GFP-tagged Stx4. Thirty-six hours after transfection, whole cell extracts were incubated with anti-myc and protein G agarose beads (Roche Diagnostics) overnight at 4 °C. The beads were washed three times with extraction buffer and resuspended in extraction buffer, and samples were analyzed by western blotting. For pulldown experiments, 293T cells were transfected with Cdo and deletion mutant of Stx4-S. Cell extracts were incubated with anti-S beads (Novagen, Madison, WI), and the precipitates were assessed by immunoblotting.

### Biotin labeling of cell surface protein

Cell surface biotinylation was performed essentially as described previously [27]. Briefly, C2C12 cells were induced to differentiate for indicated time points by switching to DM and incubating in phosphate-buffered saline (PBS) containing Sulfo-NHS-LC-Biotin (Thermo Fisher Scientific, Rockford, IL) with the final concentration of 1 mg/ml for 30 min on ice. After quenching the biotinylation, cells were lysed in extraction buffer containing protease inhibitor. Biotinylated proteins were recovered on streptavidin-agarose beads (Thermo Fisher Scientific), followed by SDS-PAGE.

### Immunocytochemistry and confocal microscopy

Immunostaining for MHC expression was performed as described previously [1]. Briefly, C2C12 cells were transfected with Cdo plus GFP vector or Stx4 and GFP vector, fixed with 4 % paraformaldehyde for 20 min, permeabilized with 1 % Triton X-100 in PBS for 10 min, blocked, and stained with anti-MHC, followed by an FITC-conjugated and Alexa Fluor 568-conjugated secondary antibody (Invitrogen). Images were captured and processed with a Nikon ECLIPSE TE-2000U microscope and NIS-Elements F software (Nikon). Quantitative differentiation assay was performed for at least three independent experiments.

For reactivation of p38 in Cdo-depleted cells by Stx4 overexpression experiment, C2C12 cells in 12-well plates were cotransfected with 100 ng of a GFP expression vector and 900 ng of the indicated DNA construct for 2 days and then fixed with 4 % paraformaldehyde for 20 min. Cultures were then permeabilized with 1 % Triton X-100 in PBS, blocked, and incubated with anti-p-p38 followed by incubation with an Alexa Fluor 568-conjugated secondary antibody. Nuclei were counterstained with 4',6-diamidino-2-phenylindole (DAPI). An image was obtained on a Zeiss LSM-510 Meta Confocal Microscope. Quantification of the fluorescent signal for p-p38 was performed with Image Gauge software (Fujifilm).

### Luciferase assay

10T1/2 cells were seeded in 12-well plates at a density of 4 × 10^4^ cells per well. Twenty-four hours after seeding, cells were transfected using Lipofectamine 2000 with 100 ng of the reporter plasmid of MyoD-luc and cotransfected with 50 ng MyoD. Twelve hours later, transfection cells were transferred into GM, harvested, and firefly luciferase activity was determined using a Luminometer with Luciferase Reporter Assay System (Promega, Fitchburg, WI). Experiments were performed in triplicates and repeated at least three times independently.

### RNA extraction, RT-PCR, and quantitative RT-PCR

Total RNA was extracted using Easy-Blue reagent (iNtRON Biotechnology, Seongnam, Korea) according to the manufacturer’s instructions. Template cDNAs were reverse-transcribed from 2 μg of total RNA using oligo-dT primer and SuperScript II reverse transcriptase (Invitrogen). The PCR mixture contained the template DNA, primer, dNTPs, and DNA polymerase (Invitrogen). PCR reactions were performed in a Genepro-PCR model. Expression levels of glyceraldehyde 3-phosphate dehydrogenase (Gapdh) were used to normalize the expression levels of each sample. Primer sequences used for PCR were as follows: Stx4, 5′-GTCTGACGAGGAGCTGGAAC-3′ and 5′-CCGAGCTCAGGATGTTCTTC-3′; Cdo intracellular region, 5′-ATAGGATCCTGGAAGAGTCGCCAACAG-3′ and 5′-ATGGTACCTCAGGTCTCTTGGGCTTG-3′; Gapdh, 5′-ATGGGGAAGGTGAAGGTCG-3′ and 5′-TTACTCCTTGGAGGCCATGT-3′. Each PCR reaction was analyzed on 1.2 % agarose gel containing ethidium bromide. Real-time PCR was performed using SYBR Green PCR master mix in an ABI cycler and quantified with ABI 7000 software (Applied Biosystems, Foster City, CA). Briefly, 1 μg of total RNAs was reverse-transcribed for 5 min at 72 °C and incubated for 5 min on ice followed by incubation for 60 min at 42 °C and 5 min at 95 °C. One hundred nanograms cDNA and 0.2 μl universal reverse (Invitrogen) and specific forward primer were used for the 20 μl PCR reaction. All PCR reactions were analyzed as triplicates.

### Measurement of glucose uptake

Stable C2C12 cells transfected with control, Cdo, or shCdo expression vector were incubated in the serum-, glucose-free DMEM for 2 h at 37 °C. After the incubation, cells were treated with 10 μg/ml insulin in the serum-, glucose-free DMEM for 1 h, and 100 μM 2-[N-(7-nitrobenz-2-oxa-1,3-diazol-4-yl) amino]-2-deoxy-D-glucose (2-NBDG) was added, a fluorescent glucose analog (Invitrogen) for 1 h. Reactions were terminated by washing with a cold DPBS buffer, followed by measurement of the fluorescence intensity at an excitation of 485 nm and an emission of 535 nm using a Luminometer (Promega).

### Statistics

The experiments were performed independently at least three times. The participants’ *t*-test was used to access the significance of the difference between two mean values. *p < 0.01 and **p < 0.05 were considered to be statistically significant.

## Results

### Stx4 is expressed in skeletal muscles and enhanced during myoblast differentiation

**Fig. 1:**
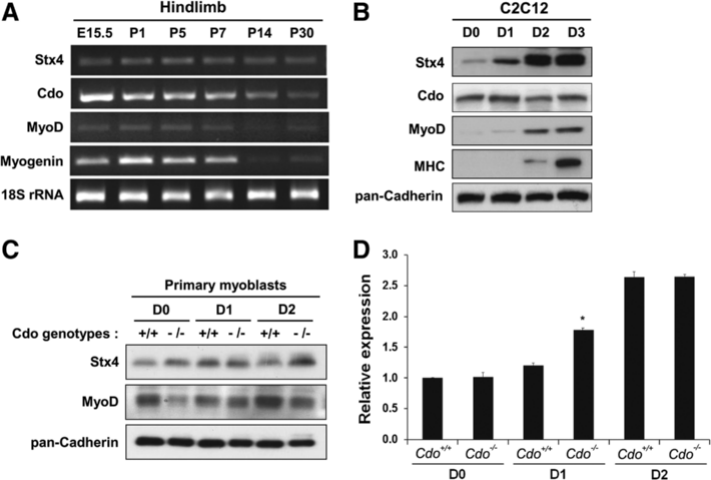
Stx4 is expressed in skeletal muscles and induced in myoblast differentiation. **a** RT-PCR analysis of hindlimb muscles from E15.5 embryos and P1, P5, P7, P14, and P30 mice for the expression of Stx4, Cdo, MyoD, Myogenin, and 18S rRNA serves as a loading control. **b** Immunoblot analysis of C2C12 cells from various differentiation days (*D*) for the expression of Stx4, Cdo, Myogenin, MHC, and pan-Cadherin serves as a loading control. **c** Immunoblot analysis for Stx4 protein expression in *Cdo* ^*+/+*^ and *Cdo* ^*−/−*^ primary myoblasts during differentiation, and pan-Cadherin serves as a loading control. **d** qRT-PCR analysis for Stx4 mRNA expression in *Cdo* ^*+/+*^ and *Cdo* ^*−/−*^ primary myoblasts during differentiation

In the previous study, we performed a yeast two-hybrid screening to identify interacting proteins for Cdo, and JLP and Bnip2 are two such proteins implicated in Cdo-mediated myogenesis [13, 14]. In the same screen, Syntaxin (Stx) 1 was identified as an interacting protein for Cdo. Stx1 and Stx4 share high homology and have similar domain structures consisting of Stx, t-SNARE, and transmembrane domain (TD) [28]. While Stx1 is expressed predominantly in neural cell types, Stx4 is the major form in skeletal muscles [29, 30]. Therefore, we examined whether Stx4 plays a role in myogenesis, especially in association with Cdo. First, we have assessed the expression pattern of Stx4 and Cdo in mouse hindlimb muscles from various developmental stages. The expression of Stx4 was detected throughout the examined stages; however, the level of Cdo, MyoD, and Myogenin decreased after the postnatal day 7 which may reflect the fast muscle growth during early postnatal life (Fig. 1a). Next, we have examined the expression pattern of Stx4 protein during myoblast differentiation. C2C12 cells were grown to near-confluency (D0) and induced to differentiate by switching to the differentiation medium for a total of 3 days (D3), followed by immunoblotting. As shown in Fig. 1b, the level of Stx4 is enhanced progressively during myoblast differentiation, while the Cdo protein is expressed throughout the differentiation time course, and the expression of Myogenin and myosin heavy chain (MHC) was dramatically enhanced at D2 or D3, respectively. These data suggest that Stx4 might be important for myoblast differentiation. Since Cdo and Stx4 were coexpressed in developing skeletal muscles, we examined the relationship between Cdo and Stx4 by using primary myoblasts isolated from *Cdo*^*+/+*^ or *Cdo*^*−/−*^ mice. Previously, we have shown that Cdo-deficient primary myoblasts display defects in myoblast differentiation and p38MAPK activation [26]. *Cdo*^*+/+*^ or *Cdo*^*−/−*^ myoblasts at high cell density (D0) were induced to differentiate by removal of basic fibroblast growth factor (bFGF) for 2 days. The expression of Stx4 in *Cdo*^*−/−*^ myoblasts was substantially increased at D2 compared to that of *Cdo*^*+/+*^ myoblasts, whereas there was only slight or no difference at D0 and D1 (Fig. 1c). In addition, the qRT-PCR analysis showed that Stx4 transcript levels were increased at D1 in Cdo-deficient myoblasts, but no difference in cells at D0 or D2 (Fig. 1d). These data suggest that the Stx4 expression level alone may not be sufficient to induce myoblast differentiation when Cdo is deficient.

### Overexpression of Stx4 enhances myogenic differentiation

**Fig. 2:**
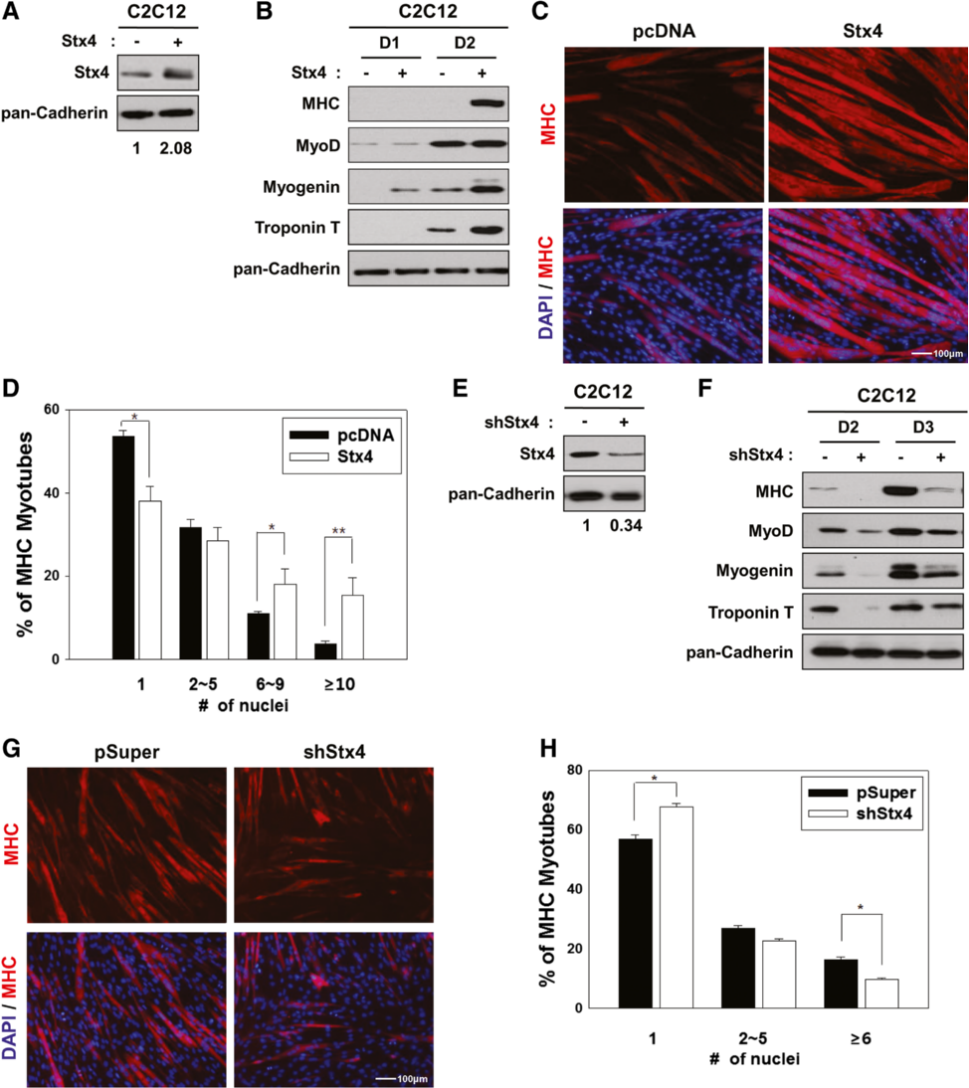
Overexpression or knockdown of Stx4 promotes or blocks myoblast differentiation, respectively. **a** Lysates of control or Stx4-overexpressing C2C12 cells were immunoblotted with antibodies against Stx4 and pan-Cadherin as a loading control. The relative signal intensity of Stx4 to pan-Cadherin was quantified and added under the blot. **b** Lysates of control or Stx4 expression vector transfected C2C12 cells from the differentiation day 1 (*D1*) and *D2* were immunoblotted with antibodies to MHC, MyoD, Myogenin, Troponin T, and pan-Cadherin as a loading control. **c** Control or Stx4 expression vector transfected C2C12 cells were induced to differentiate for 3 days and immunostained with MHC antibody followed by DAPI staining to visualize nuclei. Size bar = 100 μm. **d** The quantification of myotube formation shown in panel **c**. Values represent means of triplicate determinations ± 1 SD. The experiment was repeated three times with similar results. Significant difference from control, *p < 0.01. **e** Control or shStx4 expression vector transfected C2C12 cells were analyzed by immunoblotting with antibodies to Stx4 and pan-Cadherin as a loading control. The relative knockdown levels of Stx4 to pan-Cadherin are quantified and added under the blot. **f** Control or shStx4 expression vector transfected C2C12 cells were induced to differentiate for 2 or 3 days and then lysates were subjected to immunoblotting with antibodies to MHC, MyoD, Myogenin, Troponin T, and pan-Cadherin as a loading control. **g** Control pSuper or shStx4 expression vector transfected C2C12 cells were induced to differentiate for 3 days and immunostained with an antibody to MHC followed by DAPI staining to visualize nuclei. Size bar = 100 μm. **h** The quantification of myotube formation shown in panel **g**. Values represent means of triplicate determinations ± 1 SD. The experiment was repeated three times with similar results. Significant difference from control, *p < 0.01, **p < 0.005

To investigate the function of Stx4 in myogenesis, C2C12 cells were stably transfected with control or Stx4 expression vectors and induced to differentiate. Overexpression of Stx4 in C2C12 cells generally resulted in a twofold increase of Stx4 protein (Fig. 2a) and the expression of muscle-specific genes including MHC; Myogenin and Troponin T were significantly enhanced in Stx4-overexpressing C2C12 cells, compared to that of control cells, while MyoD levels were not altered (Fig. 2b). Next, we examined the effect of Stx4 overexpression on myotube formation. Control (pcDNA) and Stx4-overexpressing C2C12 cells were induced to differentiate for 2 days, fixed, and immunostained with anti-MHC antibody followed by DAPI staining. Stx4-overexpressing C2C12 cells formed larger myotubes than the control (pcDNA) cells (Fig. 2c, d). MHC-positive cells were scored as mononucleate, containing two to five nuclei, containing six to nine nuclei, or containing ten or more nuclei. Stx4-overexpressing cells formed more larger myotubes containing six to nine nuclei (18 %) and ten or more nuclei (15 %), compared to control cells with 10 and 3 %, respectively. In contrast, the percentile of mononucleate cells decreased to 38 %, compared to 53 % of control cells (Fig. 2d). These data suggest that Stx4 promotes myoblast differentiation.

### The depletion of Stx4 decreases myogenic differentiation

To examine whether Stx4 depletion inhibits muscle-specific gene expression and myotube formation, C2C12 cells were stably transfected with control pSuper or Stx4 shRNA (shStx4) expression vectors, induced to differentiate for 3 days and analyzed for their differentiation ability by Western blot analysis and immunostaining with anti-MHC antibody. We have tested five different Stx4 shRNA expression vectors, and among them, two shRNA constructs reproducibly resulted in a significant knockdown of Stx4. Among these, we used mostly shStx4#1 for this study (Additional file 1: Figure S1). Stx4 protein levels decreased to 34 % in C2C12/shStx4 cells, relative to that of C2C12/pSuper cells (Fig. 2e). Stx4-depleted cells exhibited a dramatic reduction in the expression of MHC, MyoD, Myogenin, and Troponin T, compared to C2C12/pSuper cells (Fig. 2f). Furthermore, C2C12/shStx4 cells formed smaller myotubes with fewer nuclei, relative to C2C12/pSuper cells (Fig. 2g). The quantification of MHC-positive cells showed that Stx depletion resulted in the formation of more mononucleated myocytes (~57 to ~67 %) and less myotubes with more than six nuclei (~17 to ~9 %), relative to C2C12/pSuper cells (Fig. 2h). These results indicate that Stx4 is required for efficient myoblast differentiation.

### Stx4 and Cdo interact physically in differentiating myoblasts, and this interaction is mediated by the t-SNARE domain of Stx4

**Fig. 3:**
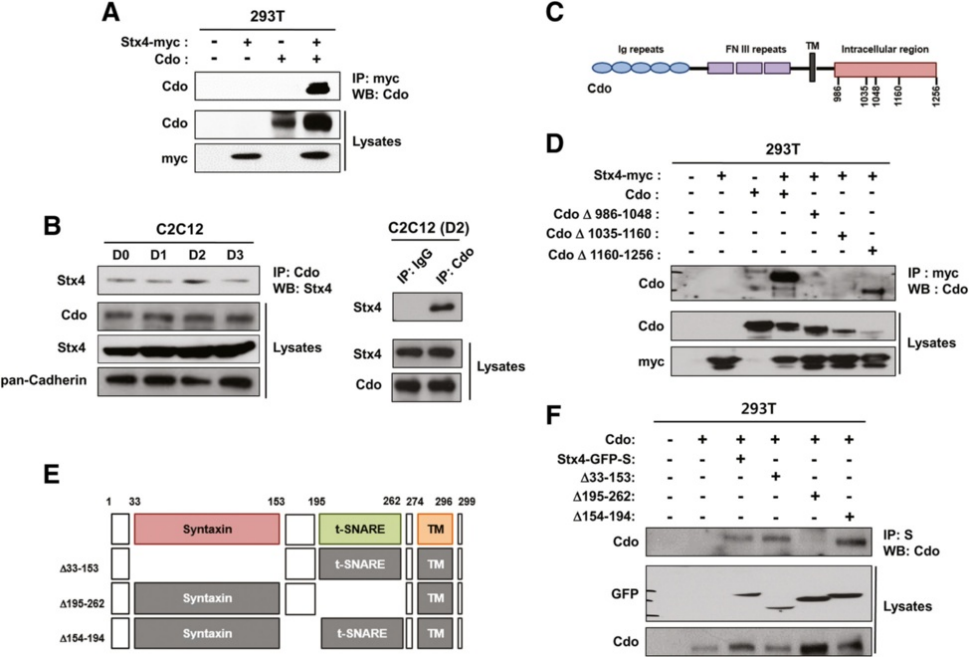
Stx4 and Cdo interact physically in differentiating myoblasts, and the t-SNARE domain of Stx4 mediates the interaction with Cdo. **a** Lysates of 293T cells transfected with Stx4-myc, Cdo, or control vector were subjected to immunoprecipitation with myc and immunoblotting with Cdo or myc antibodies. **b** Lysates of C2C12 cells from various differentiation time courses were immunoprecipitated with control IgG or anti-Cdo antibody and immunoblotted with antibodies to Stx4, Cdo, and pan-Cadherin as a loading control. **c** The schematic representation of the domain structure of Cdo. Cdo consists of five immunoglobulin, three fibronectin type III, a single transmembrane domain, and a 270-amino-acid-long intracellular region. **d** 293T cells were transiently cotransfected with control or Stx4-myc along with either the full length or three deletion mutants of the Cdo’s cytoplasmic region. Forty-eight hours later, cell lysates were subjected to immunoprecipitation with myc antibody followed by immunoblotting with Cdo antibody. Total lysates served as the expression controls. **e** The schematic representation depicts the domain structure of Stx4 and the deletion of the specific domain; Δ33-153 (the Syntaxin domain deletion), Δ195-262 (the t-SNARE region deletion), and Δ154-194 (the linker region deletion). **f** 293T cells were transiently cotransfected with control or Cdo along with the full length or deletion mutants of Stx4, and the lysates were pulled down with S-agarose beads followed by immunoblotting with Cdo or GFP antibodies

Next, we examined whether Stx4 and Cdo physically interacts in mammalian cells. To do so, 293T cells were transiently transfected with myc-tagged Stx4 and Cdo and then lysates were immunoprecipitated with the myc-tag antibody followed by immunoblotting with Cdo and myc antibodies. Consistent with the result obtained from a previous yeast two-hybrid screening [13], Stx4 and Cdo interacted in 293T cells when coexpressed (Fig. 3a). To assess whether Stx4 and Cdo interact endogenously in myoblasts, cell lysates of differentiating C2C12 myoblasts from a total of 3 days of differentiation time course were immunoprecipitated with control IgG or an anti-Cdo antibody and analyzed by Western blotting. Stx4 was precipitated with Cdo throughout differentiation time course, and the coprecipitation was highest at D2 (Fig. 3b) when myoblasts were differentiating (Fig. 1b). These results suggest that Stx4 and Cdo can physically interact in myoblasts during differentiation.

Cdo consists of an extracellular region that contains five immunoglobulin (Ig)-like repeats followed by three fibronectin type III (FNIII)-like repeats, a transmembrane segment, and a long cytoplasmic tail [31]. A schematic representation of the Cdo protein structure is shown in Fig. 3c. To determine the cytoplasmic region of Cdo which is responsible for interaction with Stx4, we have transiently transfected three Cdo mutants that harbor indicated deletions in the cytoplasmic tail and analyzed the ability of these mutants to coprecipitate with Stx4. All three Cdo mutants showed a reduction in Stx4 binding, relative to the full length. However, CdoΔ986-1048 and CdoΔ1035-1160 failed to coprecipitate Stx4 (Fig. 3d), suggesting the cytoplasmic region of Cdo is required for Stx4 interaction.

To identify the domain of Stx4 responsible for Cdo interaction, we have generated GFP-S-tagged Stx4 deletion mutants based on its domain structure (Fig. 3e). The full length or these deletion mutants of Stx4 were cotransfected into 293T cells with Cdo expression vector followed by a pulldown analysis with S-agarose beads and Western blot analysis. While Stx4Δ33-153 and Stx4Δ154-194 proteins pulled down Cdo similarly to the full-length Stx4, Stx4Δ195-262 failed to precipitate Cdo, suggesting that the t-SNARE domain (aa 195–262) of Stx4 is responsible for Cdo binding (Fig. 3f).

### The deletion mutants for either the Syntaxin or the t-SNARE domain of Stx4 failed to enhance myoblast differentiation

**Fig. 4:**
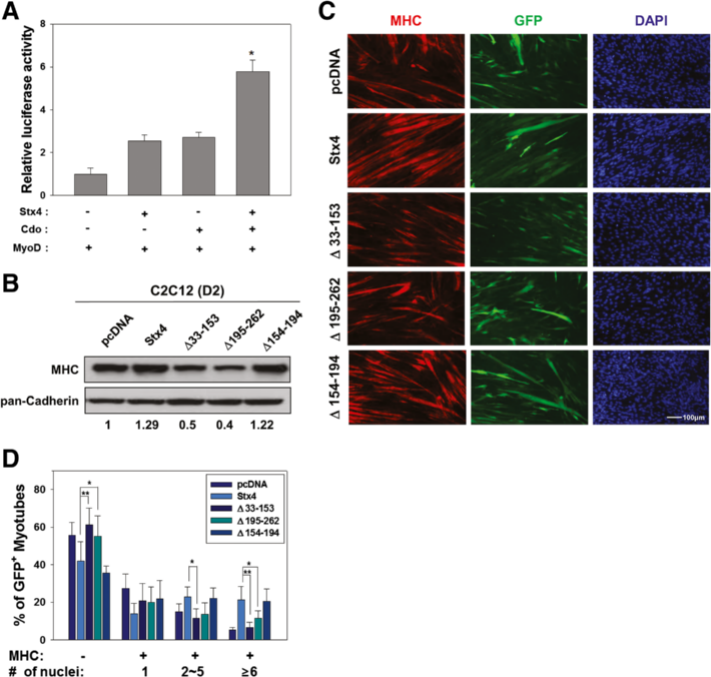
Stx4 and Cdo induce MyoD activities synergistically, and the Cdo-binding deficient Stx4 mutant failed to enhance myotube formation. **a** 10T1/2 cells were cotransfected with a MyoD-luciferase reporter and the expression vectors for MyoD and β-galactosidase as an internal control. In addition, control, Stx4, and/or Cdo expression vectors were cotransfected as indicated. Forty-eight hours later, the reporter activities were measured and normalized relative to the internal control. The experiment was performed as triplicates and repeated three times with similar results. *p < 0.01. **b** Lysates of C2C12 cells stably transfected with indicated Stx4 vectors were immunoblotted with antibodies to MHC and pan-Cadherin as a loading control. The relative signal intensities of MHC to pan-Cadherin were quantified and added under the blot. **c** C2C12 cells were transiently cotransfected with control (pcDNA), Stx4, or Stx4 mutants along with a GFP expression vector to mark the transfectant. Then, cells were induced to differentiate for 2 days, followed by immunostaining with an antibody to MHC and DAPI stain. Size bar = 100 μm. **d** Quantification of myotube formation of cell lines shown in panel **c**. Values represent means of triplicate determinations ± 1 SD. The experiment was repeated three times with similar results. Significant difference from control, *p < 0.01, **p < 0.005

The promyogenic function of Cdo involves the activation of MyoD via p38MAPK pathway [1]. Therefore, we assessed the effect of Stx4 or/and Cdo expression on MyoD activation by using a MyoD-responsive reporter. To do so, 10T1/2 fibroblasts were cotransfected with a MyoD-luciferase construct and a MyoD expression vector along with expression vectors for Stx4 and/or Cdo. Forty-eight hours later, lysates were subjected to a luciferase assay. The expression of Stx4 or Cdo singly with MyoD enhanced the luciferase activity approximately 2.5-fold and 2.7-fold, respectively, while coexpression of Stx4 and Cdo enhanced the MyoD-reporter activity to approximately 5.8-fold compared to control MyoD-expressing cells (Fig. 4a). These data suggest that Stx4 and Cdo can activate MyoD cooperatively.

To assess the functional significance of the Stx4 interaction with Cdo in myoblast differentiation, C2C12 cells stably transfected with the control, the full length, or deletion mutants of Stx4 as indicated and induced to differentiate for 2 days followed by Western blotting for MHC expression. C2C12 cells expressing either the full-length Stx4 or Stx4Δ154-194 displayed enhanced MHC expression, compared to control-vector-expressing cells. In contrast, the expression of Stx4Δ33-153 or Stx4Δ195-262 resulted in starkly decreased MHC expression (Fig. 4b). To assess the effect of Stx4 deletion mutants on myotube formation, C2C12 cells were cotransfected with the control pcDNA, the full length, or deletion mutants of Stx4 and GFP to mark transfectants and induced to differentiate for 3 days followed by immunostaining for MHC expression. Consistent with the Western blot data, the expression of Stx4Δ154-194 enhanced myotube formation to a comparable level of the full-length Stx4, as seen by fewer GFP-positive MHC-negative cells and larger GFP-positive myotubes with more nuclei per myotube, relative to control cells (Fig. 4c, d). However, roughly 60 % of the control pcDNA, Stx4Δ33-153-, or Stx4Δ195-262-expressing cells were negative for MHC expression, and a large proportion of the GFP- and MHC-positive cells were mononucleated in these cultures. These results suggest that the Syntaxin and t-SNARE domains of Stx4 are required for the promyogenic function of Stx4.

### Stx4 enhances p38MAPK phosphorylation, and Stx4 overexpression restores myoblast differentiation in Cdo-depleted cells

**Fig. 5:**
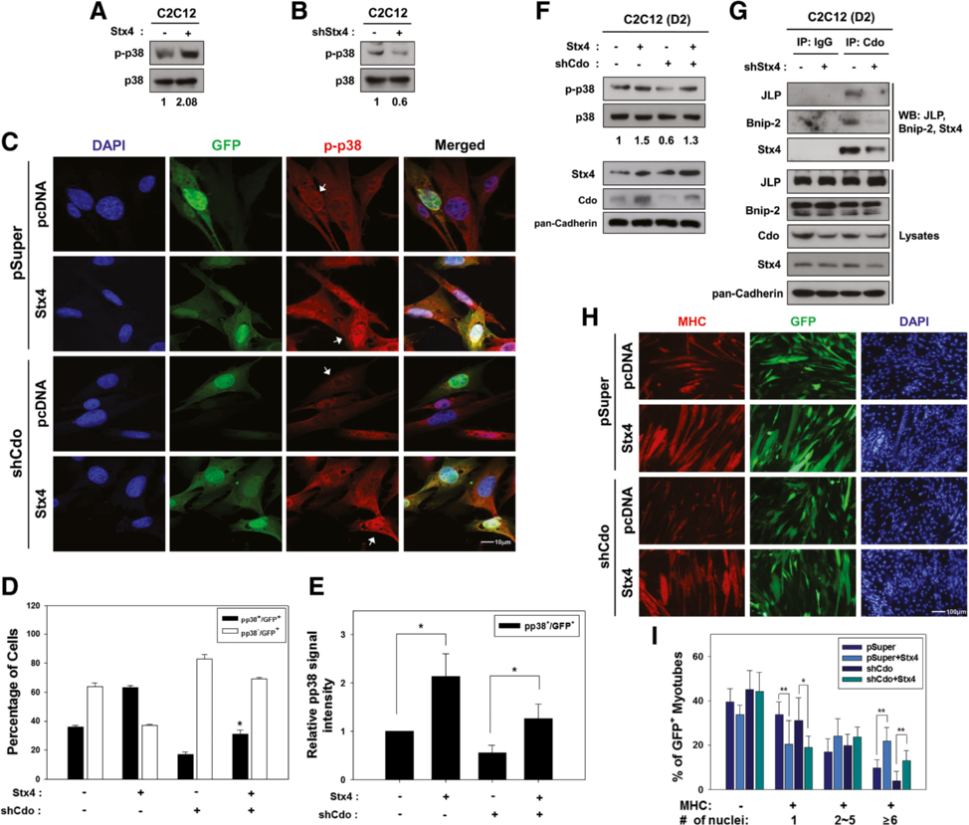
Overexpression of Stx4 can override the block of myoblast differentiation in Cdo-depleted cells. **a, b** C2C12 cells were transfected with control, Stx4, or shStx4 expression vectors, and the lysates were analyzed for the levels of phospho-p38MAPK (p-p38) relative to total p38MAPK (p38). The relative levels of p-p38 are quantified and added under each well. **c** C2C12/pSuper and C2C12/Cdo shRNA cells were transiently transfected with pcDNA or Stx4 expression vector, plus GFP expression vector to mark transfectants. Confluent cultures were then fixed and stained with antibody to p-p38 (*red*). Cell nuclei were visualized by staining with DAPI (*blue*). The *white arrows* in p-p38 panels mark the transfected cells. Size bar = 10 μm. **d** Quantification of cultures shown in **c**. GFP^+^ cells were scored as positive or negative for p-p38 staining. These experiments were repeated three times with similar results. Significant difference from control, *p < 0.01. **e** Quantification of the relative signal strength of p-p38 in GFP-positive cells. Values are determinants of 10 fields and experiments were repeated three times with similar results. *p < 0.01. **f** C2C12/pSuper and C2C12/Cdo shRNA cells were transiently transfected with pcDNA or Stx4 expression vector, and the lysates were analyzed for the levels of phospho-p38MAPK (p-p38) relative to total p38MAPK (p38). The relative levels of p-p38 are quantified and added under each well. **g** C2C12 cells were transfected with pSuper or shStx4 expression vectors and were immunoprecipitated with control IgG or anti-Cdo antibody and immunoblotted with antibodies to JLP, Bnip2, Cdo, Stx4, and pan-Cadherin as a loading control. **h** Similar sets of cells as shown in panel **c** were induced to differentiate for 3 days and immunostained for MHC and GFP expressions. Size bar = 100 μm. **i** Quantification of myotube formation from panel **e**. GFP^+^ myotubes were quantified for MHC expression and the number of nuclei present in myotubes. Values are determinants of more than 10 fields and these experiments were repeated three times with similar results. Significant difference from control, *p < 0.01, **p < 0.005

Previously, we have reported that Cdo promotes myoblast differentiation via activation of a key promyogenic kinase p38MAPK (p38) [13], and this is required for the efficient myoblast differentiation [13, 32]. Therefore, we examined the effect of Stx4 on p38 activation in C2C12 cells. Control or Stx4-overexpressing C2C12 cells were induced to differentiate for 2 days, and the status of p38 activation was analyzed by Western blot analysis with antibodies to an active phosphorylated form of p38 (p-p38) or total p38. Overexpression of Stx4 led to a substantial increase in p-p38 levels relative to that of control cells, while the level of total p38 was unchanged (Fig. 5a). In addition, C2C12/pSuper or C2C12/shStx4 cells were induced to differentiate for 3 days and analyzed for p38 activation. Stx4 knockdown in C2C12 cells caused a notable decrease in p-p38 levels relative to that of the control cells (Fig. 5b), suggesting that Stx4 is required for p38 activation during myoblast differentiation. We next asked whether the decreased p38 activation in Cdo-depleted C2C12 cells can be rescued by Stx4 expression. C2C12/pSuper and C2C12/shCdo cells were transiently transfected with pcDNA or Stx4 expression vector, plus GFP expression vector to label the transfectants. After 2 days of transfection, cells were immunostained with antibodies to p-p38 and GFP followed by DAPI staining to visualize nuclei. The representative pictures are shown in Fig. 5c. Roughly 36 % of control-transfected C2C12/pSuper cells were positive for the nuclear p-p38 accumulation (marked with a white arrow), whereas 63 % of Stx4-transfected C2C12/pSuper cells were positive for p-p38. On the other hand, only 17 % of control-transfected C2C12/shCdo cells were weakly positive for p-p38, whereas Stx4-expressing C2C12/shCdo cells displayed restored p-p38 levels with 31 % which is similar to control-transfected C2C12/pSuper cells (Fig. 5d). The quantification of relative p-p38 signal intensities in GFP-positive cells revealed the increased signal intensity in Stx4-overexpressing control cells. Furthermore, the overexpression of Stx4 restored p38 activation in C2C12/shCdo cells (Fig. 5e).

To further confirm these results, C2C12/pSuper and C2C12/shCdo cells were transfected with control or Stx4 expression vectors and induced to differentiate for 2 days, followed by Western blot analysis. As expected, C2C12/pSuper cells overexpressing Stx4 displayed elevated p-p38 levels while control transfected Cdo-depleted cells showed a reduction in p-p38 levels. In consistent with the aforementioned data, Stx4 expression in C2C12/shCdo cells restored p38 activation (Fig. 5f). Since Cdo can activate AKT via interaction with APPL1 in promotion of myoblast differentiation [15], we next examined the effect of Stx4 on AKT activation in C2C12 cells. However, the active phosphorylated AKT levels were not altered by depletion or overexpression of Stx4 (Additional file 2: Figure S2). These data suggest that Stx4 overexpression can override the block of p38MAPK activation caused by Cdo depletion in C2C12 myoblasts.

To further examine the role of Stx4 in Cdo-mediated p38 activation, we have assessed the effect of Stx4 depletion on the complex formation of Cdo with Bnip2 and JLP which has been shown to be critical for p38 activation and myogenic differentiation [13, 14]. C2C12 cells were transfected with control or shStx4 expression vector and induced to differentiate for 2 days. Cell lysates were subjected to immunoprecipitation with control IgG or anti-Cdo antibody followed by immunoblotting. The interaction of Cdo with JLP, Bnip2, and Stx4 was abrogated in Stx4-depleted cells compared to control cells (Fig. 5g). Interestingly, Cdo levels in total lysates were slightly decreased in Stx4-depleted C2C12 cells, while the levels of JLP and Bnip2 were not altered. These data suggest that Stx4 is required for Cdo/Bnip2/JLP complex formation.

This led us to investigate whether overexpression of Stx4 can restore the differentiation ability of Cdo-depleted myoblasts. C2C12/pSuper and C2C12/shCdo cells were transiently transfected with pcDNA or Stx4 plus GFP expression vectors to label the transfectants and induced to differentiate for 3 days, followed by immunostaining with a MHC antibody and DAPI staining. Consistently, Stx4 overexpression in C2C12/pSuper cells enhanced myotube formation as seen by the increased proportion of larger myotubes containing more than six nuclei compared with the control transfected cells (Fig. 5h, i). Similarly to the previous reports [26], C2C12/shCdo cells transfected with the control pcDNA exhibited impaired myotube formation. Overexpression of Stx4 in these cells restored myotube formation to similar levels of control cells (Fig. 5h, i). These results demonstrate that overexpression of Stx4 can restore the differentiation ability of Cdo-depleted C2C12 myoblasts.

### Depletion of Stx4 causes a reduction in Cdo protein levels at the cell surface

**Fig. 6:**
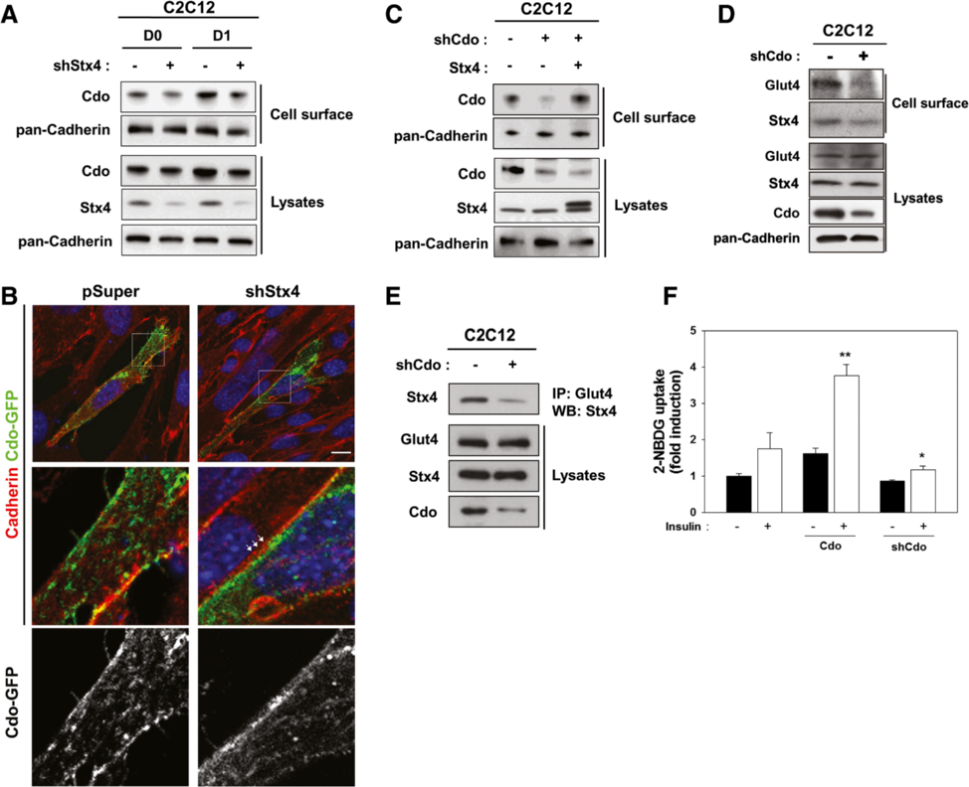
The cell-surface-resident Cdo was specifically decreased in Stx4-depleted C2C12 cells. **a** C2C12 cells were transfected with pSuper or shStx4 expression vectors, and cells at the indicated differentiation time points were subjected to the surface biotin labeling, followed by the pulldown with streptavidin and immunoblotting. Total cell lysates were also analyzed as control. **b** C2C12/pSuper or C2C12/shStx4 cells were transfected with Cdo-GFP expression vectors and subjected to immunostaining with N-Cadherin antibody, followed by confocal microscopy. The *boxed area* is shown as an enlarged view. The *white arrows* mark the area where the localization of Cdo-GFP under the N-Cadherin-resident cell surface is located. Size bar = 10 μm. **c** C2C12/pSuper or C2C12/shCdo cells were transfected with pcDNA or Stx4 expression vectors and subjected to the surface biotin labeling, followed by the pulldown with streptavidin and immunoblotting. Total cell lysates were analyzed as control. **d** Control or shCdo-transfected C2C12 cells at D1 were subjected to surface biotinylation followed by streptavidin-bead pulldown and immunoblotting with indicated antibodies. **e** Control or shCdo expression vector transfected C2C12 cells were immunoprecipitated with antibody to GLUT4 and immunoblotted with antibodies to GLUT4, Stx4, and Cdo. **f** Stable C2C12 cells transfected with control, Cdo, or shCdo expression vector were incubated with or without 10 μg/ml insulin for 1 h, followed by 2-NBDG incubation for a further 1 h. Glucose uptake was measured by the relative fluorescence intensity. The experiment was repeated for three independent assays with similar results. Significant difference from insulin-incubated cells, *p < 0.05, **p < 0.01

Next, we examined whether Stx4 regulated Cdo translocation to cell surface. To do so, we have assessed whether Stx4 depletion altered the level of Cdo at the cell surface by surface biotinylation. Stx4 knockdowned C2C12 cells displayed decreased Cdo protein levels at the cell surface as well as total Cdo proteins in lysates (Fig. 6a). Furthermore, this effect on Cdo levels appears to be specific since N-Cadherin levels did not alter in these cells. To further examine, C2C12/pSuper or C2C12/shStx4 cells were transfected with a Cdo-GFP vector and subjected to immunostaining with a Cadherin antibody to label the membrane and confocal microscopy. Cdo-GFP proteins were found at the cell membrane and intracellular compartments in both cell types. However, the signals of Cdo-GFP and Cadherin were partially superimposed at the membrane in control cells, whereas Cdo-GFP and Cadherin signals did not largely overlap at the membrane in Stx4-depleted cells (Fig. 6a). Next, we assessed whether the amount of Cdo at the cell surface is rescued by Stx4 in Cdo-depleted C2C12 cells. C2C12/shCdo cells were transfected with the Stx4 expression vector, and 24 h later, cells were analyzed by surface biotinylation. At this condition, Cdo was decreased in both pcDNA and Stx4-transfected C2C12/shCdo cells, compared to the control C2C12/pSuper cells (Fig. 6c). Consistently, the biotinylated Cdo levels were decreased in control C2C12/shCdo cells while Stx4 overexpression restored the biotinylated Cdo levels in C2C12/shCdo cells to the control C2C12/pSuper cells. These data suggest that Stx4 enhances Cdo translocation to the cell surface thereby stimulating Cdo-mediated p38 activation and myoblast differentiation.

Next, we have assessed whether Cdo is involved in GLUT4 trafficking to the cell surface mediated by Stx4. To do so, C2C12/pSuper or C2C12/shCdo cells were induced to differentiate for 1 day and analyzed for the surface biotinylation of GLUT4. The biotinylated GLUT4 levels were decreased in Cdo-depleted cells, while total GLUT4 levels did not alter (Fig. 6d). Interestingly, GLUT4 interaction with Stx4 was decreased in Cdo-depleted C2C12 cells, without affecting the total expression levels of these proteins (Fig. 6e). To assess the effect of Cdo on glucose uptake in C2C12 myoblasts, cells were stably transfected with control, Cdo, or shCdo expression vectors and treated with insulin and a fluorescent glucose analog 2-NBDG. Overexpression of Cdo in C2C12 cells generally resulted in a twofold increase of 2-NBDG uptake, while Cdo depletion reduced the level of 2-NBDG uptake to about 71 % in C2C12/shCdo cells, relative to that of control cells (Fig. 6f). Taken together, these data suggest that Stx4 regulates Cdo protein levels at the cell surface thereby enhancing the promyogenic signal triggered by Cdo, such as p38MAPK. In turn, this signaling appears to be critical for GLUT4 translocation to the cell membrane mediated by Stx4.

## Discussion

In the skeletal muscle, the role of Stx4 in glucose uptake through stimulation of GLUT4 translocation to the cell membrane in response to insulin has been well documented [19]. The fact that GLUT4 expression and translocation, likely via Stx4, exclusively induced in differentiating muscle cells [33] suggests a potential link between promyogenic signaling pathways and Stx4/GLUT4 activation to promote myoblast differentiation. Our current study suggests that Stx4 plays a critical role for myoblast differentiation. Overexpression or knockdown of Stx4 enhances or inhibits myogenic differentiation via regulation of promyogenic signaling molecules Cdo and p38. Stx4 and Cdo interact physically in differentiating myoblasts, and this interaction is mediated by the t-SNARE domain of Stx4, which is critical for the promyogenic function of Stx4. Through this interaction, Stx4 appears to regulate translocation of Cdo to the plasma membrane. It is noteworthy that Stx4 depletion results in a specific decrease in Cdo protein at the cell surface without altering N-Cadherin levels which can also interact with Cdo and promote myoblast differentiation. Vesicle transport is regulated via multiple steps including formation of vesicles or tubular intermediates, movement of vesicles towards the target compartments, tethering/docking with the acceptor membrane, and fusion of the lipid bilayers [34]. For the membrane trafficking, the specific interaction of membrane tethering and fusion is critical. Small GTPases of the Rab family are important in the stage of vesicle tethering, and SNAREs might mediate membrane fusion [34]. The mechanism how some SNAREs function in several trafficking steps and substitute for other SNAREs [35] is still unclear. In this study, we show that Cdo is a new SNARE-binding protein and directly interacts with Stx4 that regulates its cell surface localization. Considering the relatively short half-life of Cdo protein (~2 h) [36], the increased levels of Stx4 in differentiating myoblasts might ensure fast and continuous membrane trafficking of Cdo to promote myogenic differentiation. The interaction of Stx4 and Cdo can be detected already at D0 before the differentiation initiation. Considering cell adhesion signaling is an important regulator for myogenic differentiation and both Cdo and Stx4 are induced by the high cell density at D0, Cdo and Stx4 might be required for the initial stage of myogenic differentiation. In consistent with this notion, overexpression of Cdo [1] or Stx4 accelerates differentiation while depletion of these genes delays it. Thus, it is conceivable that Cdo-mediated signaling might be regulated via two sequential steps. At D0 with cell adhesion signaling, Cdo is induced and translocated to the cell surface. Subsequently, the removal of serum might relieve Cdo-mediated signaling from inhibition by growth factor signaling resulting in activation of Cdo-mediated signaling and induction of myoblast differentiation. Currently, it is unclear whether growth factor signaling directly affects Cdo-mediated signaling.

In muscle and fat cells, insulin stimulates the delivery of GLUT4 from an intracellular location to the cell surface, where it facilitates glucose uptake thereby controlling the plasma glucose levels [37]. Insulin-stimulated GLUT4 translocation seems to be regulated mainly via activation of phosphatidylinositol 3-kinase (PI3-kinase) and the AKT pathway [38]. Muscle contraction and hypoxia associated with exercise have been shown to regulate glucose uptake mainly via AMP-activated protein kinase and the CAMKII pathway [39]. In addition, p38 has been shown to regulate GLUT4 activity and glucose uptake during L6 myoblast differentiation, and inhibition of p38 reduces GLUT4 translocation and glucose uptake [33]. Previously, we have shown that the Cdo’s promyogenic function is mainly mediated by p38 which in turn activates post-translationally myogenic bHLH transcription factors, such as MyoD [26]. Stx4 overexpression and knockdown increased and reduced p38 activities, respectively (Fig. 5a, b). However, it appears that Stx4-mediated p38 activation requires Cdo, since Cdo-deficient myoblasts exhibit a decrease in the level of p-p38 and the membrane-resident GLUT4 proteins (Fig. 5c, d and Fig. 6d). Other components of Cdo-multiprotein complexes, including Boc, neogenin, and Cadherins, might be also regulated though membrane trafficking by SNARE proteins, since their expression is upregulated during myogenic differentiation [8], though Stx4 appears not to be regulating translocation of Cadherins. Interestingly, Stx4 overexpression in C2C12/shCdo cells restores the level of Cdo at the cell surface to the similar levels of the control C2C12/pSuper cells. Thus, it is likely that Stx4 might restore differentiation of Cdo-knockdown C2C12 cells via enhancing translocation of the residual Cdo. In addition, Stx4 might also regulate the translocation of other components of the Cdo-multiprotein complex such as Boc or neogenin which can stimulate Cdo-mediated myogenic differentiation. However, we cannot exclude the possibility that Stx4 may stimulate translocation of other cell membrane protein which can also activate p38 and induce myogenic differentiation. Further study will be required for elucidation of detailed mechanisms.

Cdo knockdown resulted in decreased interaction between Stx4 and GLUT4, and overexpression or knockdown of Cdo increases or decreases glucose uptake, respectively. These data suggest that Cdo-mediated p38MAPK activation may trigger Stx4 and Glut4 interaction which may trigger GLUT4 translocation to plasma membrane and glucose uptake in myoblast differentiation (Fig. 6e, f). Thus, it is conceivable that Stx4 induces translocation of Cdo first which in turn activates p38MAPK leading to the activation of Glut4 and glucose uptake during myoblast differentiation [33]. The expression of deletion mutants for the Syntaxin or t-SNARE domain responsible for Cdo binding had a dominant negative effect on MHC expression further supporting for the importance of interaction between Stx4 and Cdo and Stx4 function for its promyogenic function (Fig. 4b). It appears that the Stx4-mediated Cdo activation induces specifically p38MAPK activation since overexpression or knockdown of Stx4 did not affect the activity of another promyogenic kinase AKT which can be also upregulated by Cdo/APPL1 complexes [15]. This result suggests that the surface translocation of Cdo may not be required for AKT activation and further studies are needed to understand the detailed mechanism.

Microtubule dynamics have been shown to play an essential role in trafficking of vesicles and protein transports mediated by Stxs [40]. We have previously reported that Cdo interacts with Stim1 to regulate calcium-mediated signaling which is critical for induction of myoblast differentiation [41]. Interestingly, Stim1 can also interact with the microtubule plus end tracking protein EB1 that stabilizes the microtubule structure thereby regulating the interaction between endoplasmic reticulum and cell membrane [42]. Thus, it is plausible that Cdo localization may be regulated by a transport network involving Stim1/EB1/microtubule and Stx4 at the cell membrane. Further studies will determine whether components of vesicle trafficking and microtubule organization are involved in regulation of Cdo proteins and myogenesis. In the original yeast two-hybrid screening, Stx1B was identified as an interacting protein for Cdo [13]. Stx1 is a central component of the neuronal SNARE complex and believed to play an essential role in neurotransmitter exocytosis [17]. In addition, Stx1B has been implicated in neuronal survival [43, 44]. In our previous studies, Cdo has been shown to regulate neuronal differentiation via p38MAPK activation [45]; a similar mechanism may be applied to regulation of neuronal differentiation. The defined underlying mechanism by which Cdo and Stx1B may regulate neuronal differentiation will be addressed in the future.

## Conclusions

In conclusion, we have examined the roles of Stx4 in myoblast differentiation. Overexpression or knockdown of Stx4 enhances or inhibits myogenic differentiation, respectively, via regulation of promyogenic signaling molecules Cdo and p38MAPK. Stx4 and Cdo interact physically in differentiating myoblasts, and this interaction is mediated by the t-SNARE domain of Stx4, which is critical for the promyogenic function of Stx4. Stx4 depletion decreases specifically the cell surface resident Cdo, and the level of cell surface resident GLUT4 and interaction between GLUT4 and Stx4 are declined in Cdo-depleted cells. Therefore, interaction with Cdo and regulation of its cell surface localization by Stx4 are necessary for myogenic differentiation. In turn, this signaling appears to be critical for GLUT4 translocation to the cell membrane by Stx4 and glucose uptake.

## Additional files

Additional file 1: Figure S1. Knockdown of Stx4. Control or shStx4 expression vector transfected C2C12 cells were analyzed by immunoblotting with antibodies to Stx4 and pan-Cadherin as a loading control. The relative knockdown levels of Stx4 to pan-Cadherin is quantified and added under the blot. Additional file 2: Figure S2. Phosphorylation of AKT in Stx4-depleted or Stx4-overexpressed C2C12 cells. C2C12 cells were transfected with control, Stx4, or shStx4 expression vectors, and the lysates were analyzed for the levels of phospho-AKT (p-AKT) relative to total AKT.

## Abbreviations

Cdo: cell adhesion molecule-related, down-regulated by oncogenes
DM: differentiation medium
GLUT4: glucose transporter type 4
GM: growth medium
MHC: myosin heavy chain
p38MAPK: p38 mitogen-activated protein kinase
SNAREs: soluble *N*-ethylmaleimide-sensitive factor (NSF) attachment protein receptor proteins

## References

[1] Bae GU, Kim BG, Lee HJ, Oh JE, Lee SJ, Zhang W (2009). Cdo binds Abl to promote p38alpha/beta mitogen-activated protein kinase activity and myogenic differentiation. Mol Cell Biol.

[2] Braun T, Gautel M (2011). Transcriptional mechanisms regulating skeletal muscle differentiation, growth and homeostasis. Nat Rev Mol Cell Biol.

[3] Sartorelli V, Caretti G (2005). Mechanisms underlying the transcriptional regulation of skeletal myogenesis. Curr Opin Genet Dev.

[4] Pownall ME, Gustafsson MK, Emerson CP (2002). Myogenic regulatory factors and the specification of muscle progenitors in vertebrate embryos. Annu Rev Cell Dev Biol.

[5] Berkes CA, Tapscott SJ (2005). MyoD and the transcriptional control of myogenesis. Semin Cell Dev Biol.

[6] Ludolph DC, Konieczny SF (1995). Transcription factor families: muscling in on the myogenic program. FASEB J.

[7] Cole F, Zhang W, Geyra A, Kang JS, Krauss RS (2004). Positive regulation of myogenic bHLH factors and skeletal muscle development by the cell surface receptor CDO. Dev Cell.

[8] Krauss RS, Cole F, Gaio U, Takaesu G, Zhang W, Kang JS (2005). Close encounters: regulation of vertebrate skeletal myogenesis by cell-cell contact. J Cell Sci.

[9] Kang JS, Yi MJ, Zhang W, Feinleib JL, Cole F, Krauss RS (2004). Netrins and neogenin promote myotube formation. J Cell Biol.

[10] Leem YE, Han JW, Lee HJ, Ha HL, Kwon YL, Ho SM (2011). Gas1 cooperates with Cdo and promotes myogenic differentiation via activation of p38MAPK. Cell Signal.

[11] Kang JS, Mulieri PJ, Hu Y, Taliana L, Krauss RS (2002). BOC, an Ig superfamily member, associates with CDO to positively regulate myogenic differentiation. EMBO J.

[12] Kang JS, Feinleib JL, Knox S, Ketteringham MA, Krauss RS (2003). Promyogenic members of the Ig and Cadherin families associate to positively regulate differentiation. Proc Natl Acad Sci U S A.

[13] Takaesu G, Kang JS, Bae GU, Yi MJ, Lee CM, Reddy EP (2006). Activation of p38alpha/beta MAPK in myogenesis via binding of the scaffold protein JLP to the cell surface protein Cdo. J Cell Biol.

[14] Kang JS, Bae GU, Yi MJ, Yang YJ, Oh JE, Takaesu G (2008). A Cdo-Bnip-2-Cdc42 signaling pathway regulates p38alpha/beta MAPK activity and myogenic differentiation. J Cell Biol.

[15] Bae GU, Lee JR, Kim BG, Han JW, Leem YE, Lee HJ (2010). Cdo interacts with APPL1 and activates AKT in myoblast differentiation. Mol Biol Cell.

[16] Sollner TH (2003). Regulated exocytosis and SNARE function (review). Mol Membr Biol.

[17] Sollner T, Bennett MK, Whiteheart SW, Scheller RH, Rothman JE (1993). A protein assembly-disassembly pathway in vitro that may correspond to sequential steps of synaptic vesicle docking, activation, and fusion. Cell.

[18] Kioumourtzoglou D, Gould GW, Bryant NJ (2014). Insulin stimulates Syntaxin4 SNARE complex assembly via a novel regulatory mechanism. Mol Cell Biol.

[19] Spurlin BA, Park SY, Nevins AK, Kim JK, Thurmond DC (2004). Syntaxin 4 transgenic mice exhibit enhanced insulin-mediated glucose uptake in skeletal muscle. Diabetes.

[20] Xie L, Zhu D, Dolai S, Liang T, Qin T, Kang Y (2015). Syntaxin-4 mediates exocytosis of pre-docked and newcomer insulin granules underlying biphasic glucose-stimulated insulin secretion in human pancreatic beta cells. Diabetologia.

[21] Oh E, Stull ND, Mirmira RG, Thurmond DC (2014). Syntaxin 4 up-regulation increases efficiency of insulin release in pancreatic islets from humans with and without type 2 diabetes mellitus. J Clin Endocrinol Metab.

[22] Yang C, Coker KJ, Kim JK, Mora S, Thurmond DC, Davis AC (2001). Syntaxin 4 heterozygous knockout mice develop muscle insulin resistance. J Clin Invest.

[23] Wyman AH, Chi M, Riley J, Carayannopoulos MO, Yang C, Coker KJ (2003). Syntaxin 4 expression affects glucose transporter 8 translocation and embryo survival. Mol Endocrinol.

[24] Mitsumoto Y, Burdett E, Grant A, Klip A (1991). Differential expression of the GLUT1 and GLUT4 glucose transporters during differentiation of L6 muscle cells. Biochem Biophys Res Commun.

[25] Cole F, Krauss RS (2003). Microform holoprosencephaly in mice that lack the Ig superfamily member Cdon. Curr Biol.

[26] Tran P, Ho SM, Kim BG, Vuong TA, Leem YE, Bae GU (2012). TGF-beta-activated kinase 1 (TAK1) and apoptosis signal-regulating kinase 1 (ASK1) interact with the promyogenic receptor Cdo to promote myogenic differentiation via activation of p38MAPK pathway. J Biol Chem.

[27] Huang G, Buckler-Pena D, Nauta T, Singh M, Asmar A, Shi J (2013). Insulin responsiveness of glucose transporter 4 in 3T3-L1 cells depends on the presence of sortilin. Mol Biol Cell.

[28] Bennett MK, Garcia-Arraras JE, Elferink LA, Peterson K, Fleming AM, Hazuka CD (1993). The Syntaxin family of vesicular transport receptors. Cell.

[29] Sumitani S, Ramlal T, Liu Z, Klip A (1995). Expression of Syntaxin 4 in rat skeletal muscle and rat skeletal muscle cells in culture. Biochem Biophys Res Commun.

[30] Proux-Gillardeaux V, Galli T, Callebaut I, Mikhailik A, Calothy G, Marx M (2003). D53 is a novel endosomal SNARE-binding protein that enhances interaction of Syntaxin 1 with the synaptobrevin 2 complex in vitro. Biochem J.

[31] Kang JS, Gao M, Feinleib JL, Cotter PD, Guadagno SN, Krauss RS (1997). CDO: an oncogene-, serum-, and anchorage-regulated member of the Ig/fibronectin type III repeat family. J Cell Biol.

[32] Perdiguero E, Ruiz-Bonilla V, Gresh L, Hui L, Ballestar E, Sousa-Victor P (2007). Genetic analysis of p38 MAP kinases in myogenesis: fundamental role of p38alpha in abrogating myoblast proliferation. EMBO J.

[33] Niu W, Huang C, Nawaz Z, Levy M, Somwar R, Li D (2003). Maturation of the regulation of GLUT4 activity by p38 MAPK during L6 cell myogenesis. J Biol Chem.

[34] Zerial M, McBride H (2001). Rab proteins as membrane organizers. Nat Rev Mol Cell Biol.

[35] Rizo J, Sudhof TC (2012). The membrane fusion enigma: SNAREs, Sec1/Munc18 proteins, and their accomplices–guilty as charged?. Ann Rev Cell Dev Biol.

[36] Bae GU, Domene S, Roessler E, Schachter K, Kang JS, Muenke M (2011). Mutations in CDON, encoding a hedgehog receptor, result in holoprosencephaly and defective interactions with other hedgehog receptors. Am J Hum Genet.

[37] Bryant NJ, Govers R, James DE (2002). Regulated transport of the glucose transporter GLUT4. Nature Rev Mol Cell Biol.

[38] Nagano K, Takeuchi H, Gao J, Mori Y, Otani T, Wang D (2015). Tomosyn is a novel AKT substrate mediating insulin-dependent GLUT4 exocytosis. Int J Biochem Cell Biol.

[39] Deshmukh AS, Glund S, Tom RZ, Zierath JR (2009). Role of the AMPKgamma3 isoform in hypoxia-stimulated glucose transport in glycolytic skeletal muscle. Am J Physiol Endocrinol Metab.

[40] Du Y, Shen J, Hsu JL, Han Z, Hsu MC, Yang CC (2014). Syntaxin 6-mediated Golgi translocation plays an important role in nuclear functions of EGFR through microtubule-dependent trafficking. Oncogene.

[41] Lee HJ, Bae GU, Leem YE, Choi HK, Kang TM, Cho H (2012). Phosphorylation of Stim1 at serine 575 via netrin-2/Cdo-activated ERK1/2 is critical for the promyogenic function of Stim1. Mol Biol Cell.

[42] Grigoriev I, Gouveia S, Van der Vaart B, Demmers J, Smyth JT, Honnappa S (2008). STIM1 is a MT-plus-end-tracking protein involved in remodeling of the ER. Curr Biol.

[43] Kofuji T, Fujiwara T, Sanada M, Mishima T, Akagawa K (2014). HPC-1/Syntaxin 1A and Syntaxin 1B play distinct roles in neuronal survival. J Neurochem.

[44] Mishima T, Fujiwara T, Sanada M, Kofuji T, Kanai-Azuma M, Akagawa K (2014). Syntaxin 1B, but not Syntaxin 1A, is necessary for the regulation of synaptic vesicle exocytosis and of the readily releasable pool at central synapses. PloS ONE.

[45] Oh JE, Bae GU, Yang YJ, Yi MJ, Lee HJ, Kim BG (2009). Cdo promotes neuronal differentiation via activation of the p38 mitogen-activated protein kinase pathway. FASEB J.

