# Supplementary figures and images for: Syntaxin 4 regulates the surface localization of a promyogenic receptor Cdo thereby promoting myogenic differentiation
Source: Skelet Muscle. 2015 Sep 7;5:28. doi: 10.1186/s13395-015-0052-8 (PMC4561423; doi:10.1186/s13395-015-0052-8)

## Yoo et al., Supplementary Figure 1

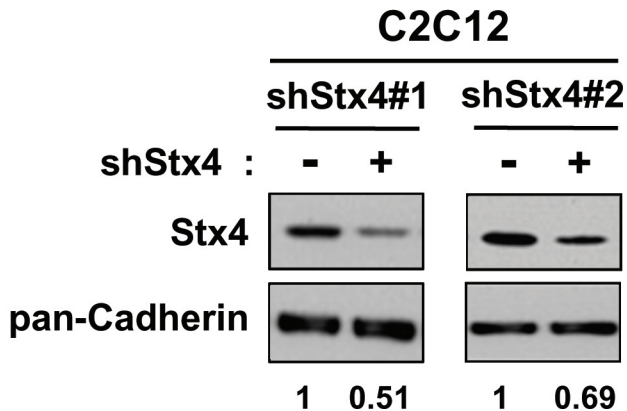

Supplement: Additional file 1: Figure S1. — Knockdown of Stx4. Control or shStx4 expression vector transfected C2C12 cells were analyzed by immunoblotting with antibodies to Stx4 and pan-Cadherin as a loading control. The relative knockdown levels of Stx4 to pan-Cadherin is quantified and added under the blot. [file 13395_2015_52_MOESM1_ESM.pdf]

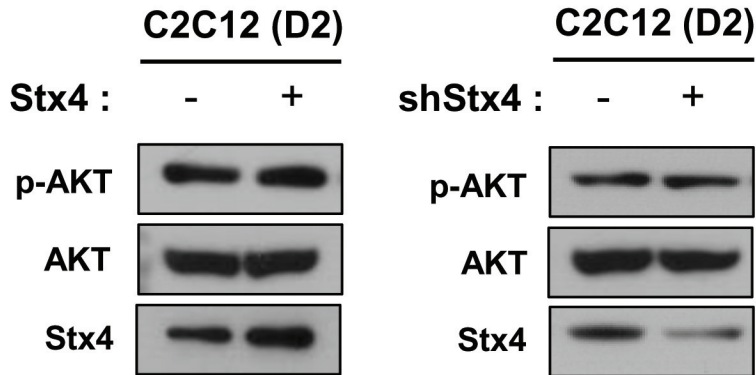

Supplement: Additional file 2: Figure S2. — Phosphorylation of AKT in Stx4-depleted or Stx4-overexpressed C2C12 cells. C2C12 cells were transfected with control, Stx4, or shStx4 expression vectors, and the lysates were analyzed for the levels of phospho-AKT (p-AKT) relative to total AKT. [file 13395_2015_52_MOESM2_ESM.pdf]
